# Herbal Medicine for Adult Patients with Cough Variant Asthma: A Systematic Review and Meta-Analysis

**DOI:** 10.1155/2021/5853137

**Published:** 2021-03-02

**Authors:** Yuan-Bin Chen, Johannah L Shergis, Zhen-Hu Wu, Xin-Feng Guo, Anthony L Zhang, Lei Wu, Fei-Ting Fan, Yin-Ji Xu, Charlie C Xue, Lin Lin

**Affiliations:** ^1^Department of Respiratory Medicine, The Second Clinical College of Guangzhou University of Chinese Medicine, The Second Affiliated Hospital of Guangzhou University of Chinese Medicine, Guangdong Provincial Hospital of Chinese Medicine, Guangzhou, China; ^2^School of Health and Biomedical Sciences, RMIT University, Bundoora, Victoria, Australia

## Abstract

**Introduction:**

Herbal medicine is commonly used by patients with chronic cough, but the role of herbal medicine for cough variant asthma (CVA) has not yet been clearly defined. For the first time, we performed a meta-analysis to integrate the current evidence of randomized controlled trials (RCTs) on this topic and assess the efficacy of herbal medicine in adults with CVA.

**Methods:**

A comprehensive search was conducted in electronic databases to identify RCTs of herbal medicine for adult CVA. Cochrane systematic review methods were followed, and the Grading of Recommendations Assessment, Development, and Evaluation was performed to evaluate the quality of evidence.

**Results:**

Twenty-eight RCTs were included. Compared with placebo, moderate-quality evidence from two studies showed that herbal medicine was associated with reduced cough symptom score (CSS) (MD −1.15 points; 95% CI, −1.67 to −0.63) and visual analogue scale (VAS) (MD −1.76 points; 95% CI, −2.66 to −0.86). Compared with montelukast, low- to moderate-quality evidence from 11 studies indicated that herbal medicine was associated with improved Leicester Cough Questionnaire (LCQ) (MD 2.38 points; 95% CI, 1.32 to 3.44), reduced CSS (SMD −0.81 points; 95% CI, −1.09 to −0.53), and VAS (MD −1.34 points; 95% CI, −1.82 to −0.86). There were no significant differences between herbal medicine and ICS plus bronchodilator.

**Conclusions:**

In adults with CVA, herbal medicine may result in improved quality of life and reduced cough frequency and severity scores compared with placebo or montelukast. Herbal medicine was not better than ICS plus a bronchodilator but the evidence is very uncertain.

## 1. Introduction

Cough variant asthma (CVA) is a form of asthma with bronchial hyperresponsiveness eosinophilic airway inflammation and airway remodeling. It presents solely with a dry or minimally productive cough, without the traditional asthma symptoms of wheezing and shortness of breath [1]. The three most common types of chronic cough, accounting for approximately 90% of cases, include CVA, upper airway cough syndrome, and gastroesophageal reflux disease [2, 3]. Moreover, patients in Asian countries experience CVA more commonly than other types of chronic cough [4, 5].

Cough variant asthma treatment is similar to typical asthma. First-line treatments should include inhaled corticosteroids (ICS) and long-acting beta-agonists, avoiding triggers [6]. If these treatments are not successful, leukotriene receptor antagonists or oral corticosteroids may be used but have limited efficacy [7]. However, the standardized treatment of CVA is limited by the following: (1) the lack of inhaled therapeutic drugs such as Advair Diskus® in community and grassroots hospitals in developing countries; (2) adverse reactions caused by ICS, for example, hoarseness, mouth ulcers, and pneumonia [8, 9]; (3) patients insensitivity or intolerance to corticosteroid therapy; and (4) people being cautious of using corticosteroids. Therefore, it is not uncommon that patients with CVA do not receive a standardized treatment plan at the beginning or are unable to adhere to the treatment, especially in China.

Although it is acknowledged that there is a lack of high-quality evidence on herbal medicine for CVA, broad-based population surveys indicate that a growing number of patients are using herbal medicine for a range of chronic diseases [10, 11]. Cough is the most common complaint when patients seek medical treatment [12], and herbal medicine may be one of the important options for many patients. In recent years, more and more clinical studies on herbal medicine for CVA have emerged. According to current Chinese national guidelines of cough, herbal medicine is considered to be effective and Suhuang Zhike capsules are recommended for CVA [13]. However, due to the absence of evidence from systematic reviews, clinicians' confidence in herbal medicine for CVA is low, and the specific role(s) of herbal medicine in the treatment of CVA is unclear. The aim of this systematic review and meta-analysis was, therefore, to evaluate the efficacy and safety of herbal medicine in adult patients with CVA.

## 2. Methods

This meta-analysis was performed using Review Manager according to the Cochrane Handbook for Systematic Reviews of Interventions (Version 5.3.3) [14] and followed the statement based on the Preferred Reporting Items for Systematic Reviews and Meta-analyses guidelines [15]. The protocol for this review was registered in PROSPERO (Identifier: CRD42018115083).

### 2.1. Search Strategy

Evidence was gathered by searching electronic English and Chinese language databases and the methods followed the Cochrane Handbook of Systematic Reviews [14]. English language databases included PubMed, Cochrane Library, Excerpta Medica Database (Embase), Cumulative Index of Nursing and Allied Health Literature, Cochrane Central Register of Controlled Trials, and Allied and Complementary Medicine Database. Chinese language databases included China BioMedical Literature, China National Knowledge Infrastructure, Chonqing VIP, and Wanfang. Databases were searched from their inception to April 2020 without language restrictions. Our search strategy was the combination of subject words and keywords including herbal medicine, CVA, and their synonyms. We also scanned reference lists of the included studies and contacted authors to acquire unpublished data.

### 2.2. Eligibility Criteria

Trials were selected based on the following inclusion criteria: (1) participants with CVA according to the American Practice Guidelines [12] or Diagnosis and Chinese Cough Guidelines [13]; (2) randomized controlled trials (RCTs) comparing herbal medicine with placebo, leukotriene receptor antagonists, or ICS plus bronchodilators; (3) trials recruiting adults older than 18 years; (4) treatment duration at least two weeks; and (5) endpoints meeting any of our prespecified outcomes of interest. Exclusion criteria were (1) trials of participants with typical asthma, (2) trials including children with CVA, and (3) trials using different pharmacotherapy cointerventions in the intervention and control groups.

### 2.3. Data Extraction

Two reviewers (YBC, JLS) independently extracted the following details from each study using EpiData software: title, authors, publication year, country of origin, patient characteristics, study design, doses and duration of intervention measures, study outcomes, and adverse events. Disagreements were resolved by discussion and consultation with a third person (ALZ). The primary outcomes were Leicester Cough Questionnaire (LCQ) [16], cough symptom score (CSS) [17, 18], and visual analogue scale (VAS). The secondary outcomes included the forced expiratory volume in one second (FEV_1_), CD4+/CD8+ ratio, effective rate [19, 20], and adverse events.

### 2.4. Risk of Bias Assessment

The risk of bias was independently evaluated according to the Cochrane Collaboration's Risk of Bias Tool [14]. Bias in each trial can be categorized into seven items including the randomization sequence generation, allocation concealment, blinding of participants and personnel, blinding of outcome assessment, incomplete outcome data, selective reporting, and other bias. Each domain was assessed to determine whether bias is at low, high, or unclear risk.

### 2.5. Statistical Analysis

We used a random-effects model to pool the available data. Estimates of heterogeneity were reported with risk ratio (RR), mean difference (MD) or standardized mean difference (SMD), and 95% confidence intervals (CIs). Formal tests for heterogeneity between summary data were performed using the *I*^*2*^ statistic. An *I*^*2*^＞50% was taken to indicate substantial heterogeneity. The sensitivity analysis was undertaken to explore potential sources of heterogeneity, based on the low risk of bias for the risk of bias domain sequence generation or studies with characteristics different from the others. Where possible and appropriate, planned subgroup analysis included duration of treatment and specific interventions to determine whether the subgroups significantly differed from one another. The statistical analysis was conducted with Review Manager version 5.3.3 software.

### 2.6. Quality of Evidence

An assessment of the strength and quality (certainty) of the evidence from RCTs was made using Grading of Recommendations Assessment, Development, and Evaluation (GRADE) [21]. The certainty of the evidence for each outcome was rated according to the outline in the GRADE approach.

## 3. Results

### 3.1. Studies Retrieved and Characteristics

**Figure 1:**
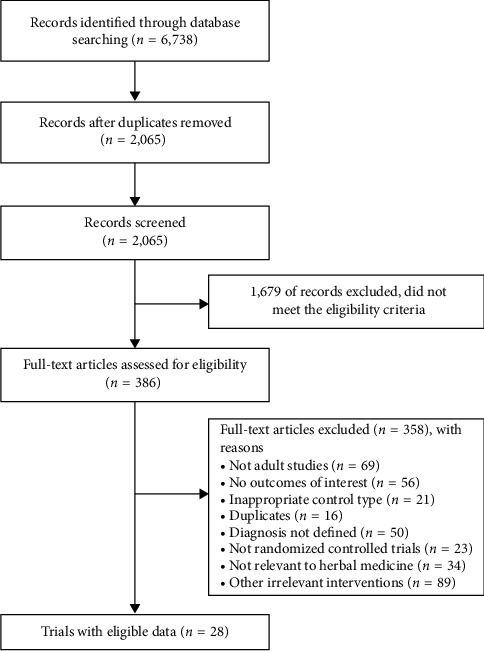
Flow chart of the study selection process.

The process of study selection is shown in Figure 1. Twenty-eight RCTs including 2,079 patients with CVA fulfilled the eligibility criteria [22–49]. All studies included two intervention arms except for four studies that included three arms; data from the two relevant arms were included in the analysis [38, 45, 48, 49].

**Figure 2:**
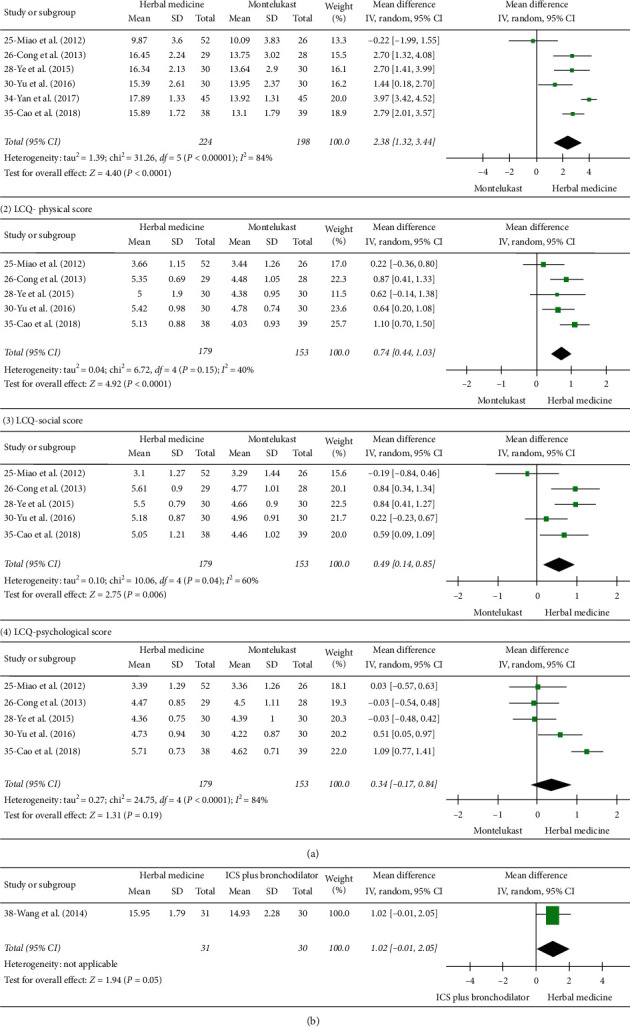
Forrest plot of Leicester Cough Questionnaire (LCQ). (a) Herbal medicine versus montelukast. (1) LCQ-total scores. (2) LCQ-physical score. (3) LCQ-social score. (4) LCQ-psychological score. (b) Herbal medicine versus ICS plus bronchodilator.

**Figure 3:**
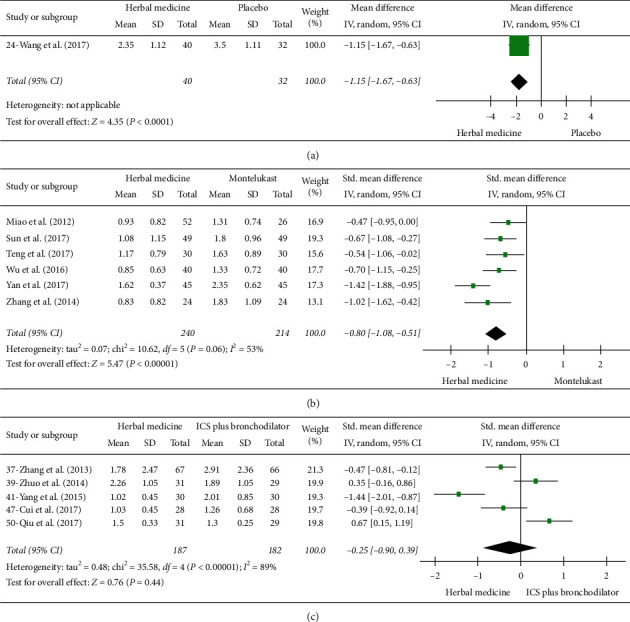
Forrest plot of cough symptom score (CSS). (a) Herbal medicine versus placebo. (b) Herbal medicine versus montelukast. (c) Herbal medicine versus ICS plus bronchodilator.

**Figure 4:**
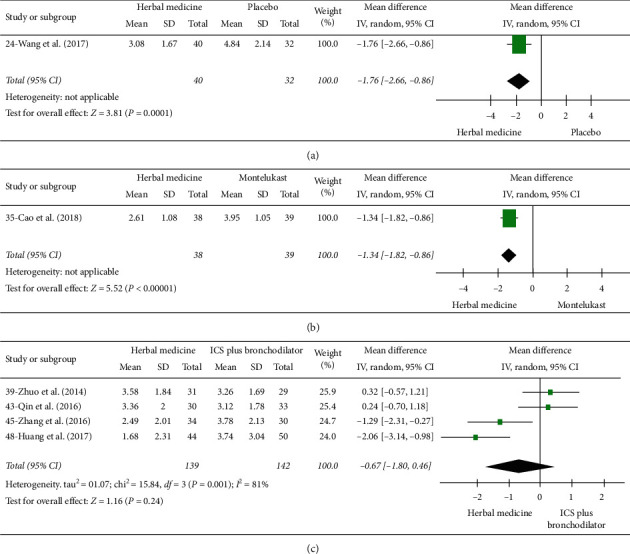
Forrest plot of visual analogue scale (VAS). (a) Herbal medicine versus placebo. (b) Herbal medicine versus montelukast. (c) Herbal medicine versus ICS plus bronchodilator.

**Figure 5:**
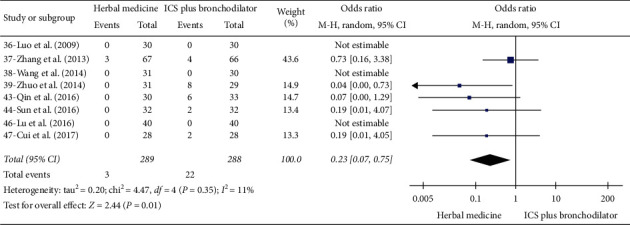
Incidence of adverse events in herbal medicine versus ICS plus bronchodilator.

Characteristics of the studies are summarized in Table 1. The 28 studies were divided into three groups for analysis: (1) herbal medicine versus placebo, (2) herbal medicine versus leukotriene receptor antagonists, and (3) herbal medicine versus ICS plus bronchodilator. Results for each grouping are presented in the GRADE summary of findings tables (Tables S1–S3) and meta-analysis forest plots (Figures 2–5 and Figures S2–S5).

Chinese medicine syndrome classification was used in 19 studies [23–25, 27, 29, 30, 32–35, 37, 39, 42–48]. The most frequently reported syndrome was severe wind attacking the lungs (8 studies) [23, 25, 30, 32, 35, 45–47]. All formulae of herbal medicine were administered orally, with decoction, granule powder, or capsules. In 11 studies [24–34], montelukast 10 mg was given as a comparator. In the studies that used ICS plus bronchodilator as a comparator, salmeterol and fluticasone propionate were used in 10 studies [35, 37, 39–42, 44–46, 48], budesonide and formoterol fumarate in four studies [38, 43, 47, 49], and budesonide and salbutamol in one study [36]. The duration of treatment ranged from 2 to 12 weeks.

### 3.2. Risk of Bias

All studies specified that “randomization” was used in the allocation of participants to the herbal medicine intervention or the control groups. However, 42.9% of studies (*n* = 12) did not clearly specify the method of random sequence generation [27, 28, 30, 31, 35, 38, 39, 43, 44, 46, 47, 49]. Three studies described an adequate method of allocation concealment and were assessed as low risk of bias [22, 23, 37]. Two studies were evaluated as low risk of bias in the blinding of participants and personnel because a placebo of herbal medicine was used as the comparator [22, 23]. Other studies lacked information about blinding of participants and personnel and were at high risk of bias. Two studies specified the information of blinding of outcome assessors [22, 23] and were assessed as low risk. The majority (89.3%) were at low risk because there was no missing data or dropouts were balanced between groups [22–41, 43–48]. One study did not report its predefined primary outcome without reason and was at high risk of reporting bias. The risk of bias is summarized in Figure S1.

### 3.3. Results of Herbal Medicine versus Montelukast

#### 3.3.1. Leicester Cough Questionnaire (LCQ)

Low-quality evidence from six studies used the LCQ to assess health-related quality of life in 422 participants (Table S1) [24, 25, 27, 29, 33, 34]. The result indicated that participants receiving herbal medicine had significantly increased LCQ-total scores (indicating reduced symptoms) than those receiving montelukast, and the difference achieved the minimal clinically important difference of 1.3 points [50], although the heterogeneity was substantial (MD 2.38 points; 95% CI, 1.32 to 3.44; *p* < 0.00001, *I*^*2*^ = 84%) (Figure 2).

The LCQ that comprises three domains (physical, psychological, and social) was available in five studies. The physical score increased in the herbal medicine group more than the montelukast group, heterogeneity might not be important (MD 0.74 points; 95% CI, 0.44 to 1.03; *p* < 0.00001, *I*^*2*^ = 40%), and social score also improved (MD 0.49 points; 95% CI, 0.14 to 0.85; *p*=0.0006, *I*^*2*^ = 60%). However, there was no difference between groups in terms of psychological score (MD 0.34 points; 95% CI, −0.17 to 0.84; *p*=0.19, *I*^*2*^ = 84%) (Figure 2).

#### 3.3.2. Cough Symptom Score (CSS)

Low-quality evidence from six studies (454 participants) evaluated CSS (Table S1) [24, 26, 28, 31–33]. The pooled result indicated that herbal medicine was superior to montelukast, and the heterogeneity was moderate (SMD −0.80 points; 95% CI, −1.08 to −0.51; *p* < 0.00001, *I*^*2*^ = 53%) (Figure 3).

#### 3.3.3. Visual Analogue Scale (VAS)

Low-quality evidence from one RCT with 77 participants assessed cough VAS using a numerical rating scale (Table S1) [34]. The cough was reduced in participants receiving herbal medicine compared to those receiving montelukast (MD −1.34 points; 95% CI, −1.82 to −0.86) (Figure 4).

#### 3.3.4. Forced Expiratory Volume in One Second (FEV1)

Low-quality evidence from two studies (148 participants) assessed FEV_1_ (Table S1). One study reported FEV_1_ liters (*L*) [30], and one reported FEV_1_ percentage (%) [26]. FEV_1_ (*L*) increased in participants receiving herbal medicine compared to montelukast (MD 0.29 L; 95% CI, 0.07 to 0.51), and FEV_1_% also improved (MD 2.84%; 95% CI, 0.35 to 5.33) (Figure S3).

#### 3.3.5. Effective Rate

Moderate-quality evidence from 10 studies [24–26, 28–34], including 748 participants, assessed the effective rate (Table S1). The effective rate was classified as the number of people with improved symptoms. Treatments were classified as effective in people with clinically controlled or markedly improved symptoms, and treatments were considered ineffective when people only reported a limited improvement or no symptom improvement. The overall result showed an improvement in the herbal medicine group compared to the montelukast group, and heterogeneity was moderate (RR 1.48; 95% CI, 1.29 to 1.71; *p* < 0.00001, *I*^*2*^ = 44%) (Figure S4).

### 3.4. Results of Herbal Medicine versus ICS plus Bronchodilator

#### 3.4.1. Leicester Cough Questionnaire (LCQ)

Low-quality evidence from one study of 61 participants showed that there was no difference between herbal medicine and ICS plus bronchodilator groups (MD 1.02 points; 95% CI, −0.01 to 2.05) (Figure 2 and Table S2) [37].

#### 3.4.2. Cough Symptom Score (CSS)

Low-quality evidence from five studies with 369 participants assessed CSS (Table S2) [36, 38, 40, 46, 49]. The overall result indicated that participants receiving herbal medicine were not better than those receiving ICS plus bronchodilator, and heterogeneity was substantial (SMD −0.25 points; 95% CI, −0.90 to 0.39; *p*=0.44, *I*^*2*^ = 89%) (Figure 3).

#### 3.4.3. Visual Analogue Scale (VAS)

Cough VAS was available in four studies which were judged as very low-quality evidence (281 participants) (Table S2) [38, 42, 44, 47]. The mean change in cough VAS was not statistically different between herbal medicine and ICS plus bronchodilator groups, and heterogeneity was considerable (MD −0.67 points; 95% CI, −1.80 to 0.46; *p*=0.24, *I*^*2*^ = 81%) (Figure 4).

#### 3.4.4. Forced Expiratory Volume in One Second (FEV1)

ICS plus bronchodilator was used as a comparator in four RCTs with low-quality evidence (*n* = 287) evaluating FEV_1_ percent (Table S2) and one RCT (*n* = 118) evaluating FEV_1_ liters [41, 43, 45, 48]. FEV_1_% increased in participants receiving herbal medicine compared to ICS plus bronchodilator, and heterogeneity might not be important (MD 3.83%; 95% CI, 1.55 to 6.10; *p*=0.001, *I*^*2*^ = 36%), but herbal medicine was not superior to ICS plus bronchodilator in terms of FEV_1_ (*L*) in one study (MD −0.01 L; 95% CI, −0.12 to 0.10) (Figure S3).

#### 3.4.5. Effective Rate

Low-quality evidence from seven studies with 531 participants evaluated effective rate (Table S2) [35, 39–42, 46, 47]. The overall result showed that participants receiving herbal medicine were not better than those receiving ICS plus bronchodilator, and heterogeneity was substantial (RR 1.21; 95% CI, 1.00 to 1.47; *p*=0.05, *I*^*2*^ = 55%). Subgroup analysis was conducted on studies that had a duration of treatment less than four weeks or greater than four weeks. There was no significant advantage with a short treatment duration or longer treatment duration (RR 1.30; 95% CI, 0.82 to 2.08; *p*=0.27, *I*^*2*^ = 70% and RR 1.14; 95% CI, 0.95 to 1.37; *p*=0.15, *I*^*2*^ = 36%, respectively) (Figure S4).

### 3.5. Results of Herbal Medicine versus Placebo

Two studies compared herbal medicine with placebo [22, 23]. Moderate-quality evidence from one study indicated that herbal medicine was superior to placebo and decreased the CSS (MD −1.15 points; 95% CI, −1.67 to −0.63) and VAS (MD −1.76 points; 95% CI, −2.66 to −0.86) (Figures 3 and 4 and Table S3) [23]. Participants receiving herbal medicine had an improved effective rate compared to participants taking placebo (RR 2.86; 95% CI, 1.42 to 5.74) (Figure S4 and Table S3). Moderate-quality evidence from one study showed that immune function in terms of CD4+/CD8+ ratio in participants receiving herbal medicine was better than the placebo group (MD −0.54; 95% CI, −0.73 to −0.35) (Figure S2 and Table S3) [22].

### 3.6. Sensitivity Analysis

In the sensitivity analysis for herbal medicine versus montelukast, four studies [27, 28, 30, 31] were excluded from the main analysis because they were identified as unclear risk of random sequence generation, and the overall results of LCQ and CSS did not change. One study [43] was excluded in the sensitivity analysis because of unclear risk of selection bias; the result indicated that herbal medicine was not superior to ICS plus bronchodilator in terms of FEV_1_ (%) in three studies (MD 3.66; 95% CI, −0.58 to 7.89, *I*^*2*^ = 50%) (Figure S5) [41, 45, 48].

### 3.7. Safety and Adverse Events

Out of the 28 studies, 14 mentioned adverse events [22, 26, 28, 29, 31, 34–38, 42, 43, 45, 46]. In one study that compared herbal medicine with placebo, there were no adverse events reported [22]. Five studies comparing herbal medicine with montelukast reported eight adverse events [26, 28, 29, 31, 34]. Constipation (2 cases) and diarrhea (2 cases) were reported in participants taking herbal medicine, while nausea (2 cases) and diarrhea (2 cases) were reported in the montelukast group. The incidence of any adverse event from eight studies was significantly higher in the participants receiving ICS plus bronchodilator than in those receiving herbal medicine (OR 0.23; 95% CI, 0.07 to 0.75; *p*=0.01, *I*^*2*^ = 11%) (Figure 5) [35–38, 42, 43, 45, 46]. Only three events were reported in the herbal medicine group, including abdominal distension (2 cases) and nausea (1 case), but 22 adverse events were reported in participants taking ICS plus bronchodilator. Events included pharyngeal discomfort (11 cases), hoarseness of voice (8 cases), nausea (1 case), palpitation (1 case), and tremor (1 case).

## 4. Discussion

The systematic review included 28 RCTs involving 2,079 adult patients with CVA. Compared with placebo, herbal medicine reduced cough intensity and improved immune function, with a moderate level of certainty. Moderate- to low-quality evidence suggested that herbal medicine may also be associated with reduced cough symptoms and improved quality of life and FEV_1_ when compared with montelukast. However, herbal medicine was not superior to ICS plus bronchodilator, with a very low to a low level of certainty. The incidence and severity of adverse events were similar between montelukast and herbal medicine. Compared with ICS plus bronchodilator, relatively few adverse events from herbal medicine were reported, and ICS plus bronchodilator was associated with increased pharyngeal discomfort and hoarseness of voice. The meta-analysis provides an up-to-date analysis of herbal medicine for the treatment of adult CVA.

Cough variant asthma (CVA) features include airway hyperresponsiveness, atopy, and airway remodeling, as well as eosinophilic airway inflammation [51]. The efficacy of montelukast in treating cough due to asthma is related to its inhibition of inflammatory mediators such as leukotrienes [52]. The importance of leukotriene receptor antagonist was demonstrated in several guidelines, and systemic leukotriene antagonists such as montelukast in CVA have greater efficacy than classical asthma [53]. In the ACCP evidence-based guidelines, the leukotriene receptor antagonist is applied in a subgroup of patients whose cough had been refractory to therapy with inhaled steroids (quality of evidence, low; grade of recommendation, B) [12]. The ERS guidelines suggest a short-term antileukotriene trial (2–4 weeks) in adult patients with asthmatic cough (conditional recommendation, low-quality evidence) [54]. The leukotriene receptor antagonist is also recommended by Chinese guidelines because it reduces cough symptoms and airway inflammation (grade of recommendation, B) [13].

We found an improved association with the benefits of oral herbal medicine (for 2–4 weeks) in reducing cough in terms of physical and social scores on the LCQ over montelukast. The results also showed potential benefits of herbal medicine in reducing subjective cough frequency and severity scores, as well as improving lung function. Moreover, in terms of safety, herbal medicine was similar to montelukast. Herbal medicine as a monotherapy is more effective with regard to clinical outcomes than montelukast, although this finding was based on moderate- to low-quality evidence.

The positive results from two double-blind, placebo-control studies also suggest the potential efficacy of herbal medicine in improving subjective cough scores and regulating immune function.

Almost all clinical practice guidelines recommend ICS plus bronchodilator as the standard treatment for CVA [12, 13, 54, 55]. Treatment is expected to be effective in the short term (within 2–4 weeks) [12, 54, 55], except Chinese guidelines that suggested the duration of treatment should be more than eight weeks and some patients require long-term treatment [13]. The standardized treatment usually has a good effect, especially corticosteroids because eosinophils are implicated in CVA and corticosteroids reduce eosinophilic inflammation [51]. However, there are some patients with CVA that are refractory to treatment with ICS plus bronchodilator. Furthermore, a reduced association with a clinical benefit over time might lead to the prescription of long-term or higher corticosteroid doses and consequently more risk of adverse events.

Our review revealed that herbal medicine did not significantly improve subjective cough outcomes and quality of life compared to ICS plus bronchodilator, but the risk of adverse events due to ICS plus bronchodilator was seven times higher than herbal medicine. Because of the possibly better long-term safety profile of herbal medicine, it may be considered as an add-on treatment alternative in CVA in suboptimally controlled patients taking ICS plus bronchodilator. Where medical decision-making is concerned, we should not only pay attention to the availability of cough control therapies and the patient's response in terms of symptom relief but also consider the side effects.

Herbal medicine has therapeutic effects largely attributable to its active compounds. Experimental evidence including *in vitro* and *in vivo* studies helps to explain the possible mechanisms of action of the herbs and how they may improve the signs and symptoms of chronic cough. There are still a limited number of CVA models, and the majority of experimental studies focus on surrogate outcomes such as anti-inflammation, antioxidation, antiallergy, or immunomodulation [56–58]. Many antitussive herbs including *Gan cao* (*Glycyrrhiza* spp.) [59], Ma *huang* (*Ephedra sinica* Stapf) [60], and *Chan tui* (*Cryptotympana pustulata* Fabricius) [61] are likely to have antispasmodic or bronchodilator actions, although these effects are yet to be explored in experimental models. The research indicates that herbal medicine has important actions that interact and modulate the key pathophysiological mechanisms associated with CVA. Potentially, there are additional benefits of the herbs on associated pathogenesis that have not been fully researched in animal models of cough but show promise.

### 4.1. Limitations

This review had several limitations. First, there was considerable heterogeneity among the included trials in some outcome measures. Second, the results could not demonstrate the differences between the various leukotriene receptor antagonists and herbal medicines, because the studies did not include other antileukotriene drugs except for montelukast, and the efficacy and safety profiles may be not the same. Third, the evaluation of CVA is mainly based on subjective outcomes, but most of the included studies were open-label trials; therefore, bias cannot be ruled out. Finally, the methodological quality was low to moderate, and the results of this meta-analysis should be interpreted with caution because none of the included studies were free from bias.

### 4.2. Suggestions

What is the role of herbal medicine in adult patients with CVA? Current guidelines lack specific recommendations as to how and when herbal medicine should be used for patients with CVA. The results of our review suggest that herbal medicine could be considered in the following situations: (1) in patients who did not respond or had a suboptimal response to ICS plus bronchodilator treatments, (2) in patients who did not respond or had a suboptimal response to montelukast, and (3) in patients who have a strong preference to take herbal medicine.

## 5. Conclusions

In this meta-analysis of RCTs of adult patients with CVA, herbal medicine was superior to placebo and montelukast but not ICS plus bronchodilator, based on very low- to moderate-level evidence. Herbal medicine was safe and associated with a lower incidence of adverse events compared with ICS plus bronchodilator. These findings indicate that people with chronic cough may benefit from herbal medicine. As new research emerges, specific recommendations for their use should be updated, especially in countries where herbal medicine is a significant part of the health care system such as in China.

## Figures and Tables

**Table 1:** Characteristics of the included studies.

Author (year)	Study design	Number of participants (I/C)	Age, years (mean, standard deviation)	Gender (M/F) (%)	Intervention/dose/frequency	Control/dose/frequency	Treatment duration (weeks)	Outcomes ^*∗*^
Zhang et al. (2009) [23]	Randomized, double-blind, placebo-controlled	74 (36/38)	I: 33.42 ± 11.26 C: 34.35 ± 10.90	51.4/48.6	Yu-ping-feng san, twice a day	Placebo, twice a day	8	⑤⑦
Wang et al. (2017) [24]	Randomized, double-blind, placebo-controlled	80 (40/40)	18–75	29.2/70.8	Qu-feng-xuan-fei formula, one dose a day	Placebo, one dose a day	2	②③
Miao et al. (2012) [25]	Randomized, open-label trial	84 (56/28)	I: 45.81 ± 13.31 C: 43.85 ± 11.45	31.0/69.0	Self-designed formula, one dose a day	Montelukast 10 mg, once daily	4	①②⑥
Cong et al. (2013) [26]	Randomized, open-label trial	60 (30/30)	I: 47.41 ± 13.34 C: 45.57 ± 13.34	35.1/64.9	Wen-run-xin-jin fang, one dose a day	Montelukast 10 mg, once daily	2	①⑥
Zhang et al. (2014) [27]	Randomized, open-label trial	48 (24/24)	I: 41.17 ± 13.69 C: 41.25 ± 14.01	37.5/62.5	Hua-gai san, one dose a day	Montelukast 10 mg, once daily	2	②④⑥⑦
Ye et al. (2015) [28]	Randomized, open-label trial	60 (30/30)	I: 43.37 ± 3.34 C: 44.00 ± 3.37	40.0/60.0	Wen-dan formula, once daily	Montelukast 10 mg, once daily	4	①
Wu et al. (2016) [29]	Randomized, open-label trial	80 (40/40)	I: 43.63 ± 8.74 C: 39.52 ± 9.16	52.5/47.5	Bu-zhong-yi-qi formula, one dose a day	Montelukast 10 mg, once daily	8	②⑥⑦
Yu et al. (2016) [30]	Randomized, open-label trial	60 (30/30)	I: 35.30 ± 10.77 C: 37.93 ± 12.69	41.7/58.3	Qu-feng-xuan-fei formula, one dose a day	Montelukast 10 mg, once daily	4	①⑥⑦
Kang et al. (2017) [31]	Randomized, open-label trial	100 (50/50)	I: 35.60 ± 6.20 C: 34.70 ± 7.80	54.0/46.0	Chan-yi-he-ji, one dose a day	Montelukast 10 mg, once daily	4	④⑥
Sun et al. (2017) [32]	Randomized, open-label trial	98 (49/49)	I: 45.51 ± 5.36 C: 47.61 ± 5.62	51.0/49.0	Su-huang-zhi-ke capsules, 3 capsules, 3 times a day	Montelukast 10 mg, once daily	2	②⑥⑦
Teng et al. (2017) [33]	Randomized, open-label trial	60 (30/30)	I: 53.77 ± 6.63 C: 52.87 ± 4.89	38.3/61.7	Zhi-ke-ping-chuan formula, one dose a day	Montelukast 10 mg, once daily	2	②⑥
Yan et al. (2017) [34]	Randomized, open-label trial	90 (45/45)	I: 35.62 ± 6.55 C: 35.51 ± 6.65	48.9/51.1	Yi-qi-qu-feng formula, one dose a day	Montelukast 10 mg, once daily	4	①②⑥
Cao et al. (2018) [35]	Randomized, open-label trial	80 (40/40)	I: 69.68 ± 4.54 C: 71.03 ± 4.67	54.6/45.4	Jia-wei-ma-xing-er-chen formula, one dose a day	Montelukast 10 mg, once daily	4	①③⑥⑦
Luo et al. (2009) [36]	Randomized, open-label trial	60 (30/30)	I: 41.97 ± 10.97 C: 43.43 ± 9.51	55.0/45.0	Ke-ping formula, one dose a day	Seretide 50/100 *μ*g, twice a day	4	⑥⑦
Zhang et al. (2013) [37]	Randomized, open-label trial	140 (70/70)	I: 37.40 ± 11.20 C: 38.10 ± 10.50	39.8/60.2	Gu-ben-zhi-ke granule, one dose a day	Budesonide 0.2 mg plus salbutamol 200 *μ*g, twice a day	8	②⑦
Wang et al. (2014) [38]	Randomized, open-label trial	62 (31/31)	I: 45.71 ± 12.28 C: 44.43 ± 11.61	42.6/57.4	Wen-dan formula, once daily	Seretide 50/250 *μ*g, twice a day	2	①⑦
Zhuo et al. (2014) [39]	Randomized, three-arms, open-label trial	93 (31/29)	I: 35.10 ± 10.90 C: 38.20 ± 11.00	57.0/43.0	Su-huang-zhi-ke capsules, 3 capsules, 3 times a day	Symbicort Turbuhaler 160/4.5 *μ*g, twice a day	4	②③⑦
Xin et al. (2015) [40]	Randomized, open-label trial	118(60/58)	22–66	53.4/46.6	Jiu-xian san, once daily	Seretide 50/250 *μ*g, twice a day	2	④⑥
Yang et al. (2015) [41]	Randomized, open-label trial	60 (30/30)	I: 45.85 ± 13.76 C: 47.02 ± 12.58	33.3/66.7	Self-designed formula, once daily	Seretide 50/100 *μ*g, twice a day	8	②⑥
Zhao et al. (2015) [42]	Randomized, open-label trial	92 (46/46)	I: 52.68 ± 16.42 C: 49.35 ± 14.27	52.2/47.8	Yi-qi-bu-shen formula, once daily	Seretide 50/250 *μ*g, twice a day	8	④⑥
Qin et al. (2016) [43]	Randomized, open-label trial	63 (30/33)	I: 39.23 ± 12.41 C: 38.63 ± 13.87	41.3/58.7	Chai-hu-zhi-ju formula, once daily	Seretide 50/250 *μ*g, twice a day	2	③⑥⑦
Sun et al. (2016) [44]	Randomized, open-label trial	64 (32/32)	I: 34.19 ± 11.09 C: 33.84 ± 11.43	51.6/48.4	Yang-yin-qu-feng formula, once daily	Symbicort Turbuhaler 160/4.5 *μ*g, twice a day	4	④⑦
Zhang et al. (2016) [45]	Randomized, open-label trial	71 (38/31)	I: 37.24 ± 1.78 C: 39.73 ± 2.42	36.0/64.0	Qu-feng-hua-tan formula, once daily	Seretide 50/250 *μ*g, twice a day	4	③
Lu et al. (2016) [46]	Randomized, three-arms,, open-label trial	120 (40/40)	I: 46.23 ± 12.59 C: 44.00 ± 13.79	30.8/69.2	Shu-feng-zhi-sou formula, once daily	Seretide 50/250 *μ*g, twice a day	4	④⑦
Cui et al. (2017) [47]	Randomized, open-label trial	60 (30/30)	I: 45.63 ± 13.67 C: 47.21 ± 14.42	48.3/51.7	Bu-shen-qu-feng-zhi-ke formula, once daily	Seretide 50/250 *μ*g, twice a day	8	②⑥⑦
Huang et al. (2017) [48]	Randomized, open-label trial	102 (50/52)	I: 32.79 ± 6.58 C: 34.21 ± 8.58	48.9/51.1	Qu-feng-zhi-ke formula, once daily	Symbicort Turbuhaler 80/4.5 *μ*g, twice a day	2	③⑥
Lu et al. (2017) [49]	Randomized, three-arms, open-label trial	90 (30/30)	I: 42.70 ± 13.35 C: 43.98 ± 11.70	28.9/71.1	Tiao-zhong-yi-fei formula, once daily	Seretide 50/250 *μ*g, twice a day	12	④
Qiu et al. (2017) [50]	Randomized, three-arms	98 (31/29)	30.10 ± 8.20	49.0/51.0	Su-huang-zhi-ke capsules, 3 capsules, 3 times a day	Symbicort Turbuhaler 160/4.5 *μ*g, twice a day	4	②

^*∗*^Outcome measures included ①Leicester Cough Questionnaire; ②cough symptom score; ③visual analogue scale; ④forced expiratory volume in one second; ⑤immune function; ⑥effective rate; ⑦adverse events.

## Data Availability

All the data of this review are available from the public, open-access electronic databases.

## Abbreviations

CSS: Cough symptom score
CVA: Cough variant asthma
FEV1: Forced expiratory volume in one second
GRADE: Grading of Recommendations Assessment, Development, and Evaluation
ICS: Inhaled corticosteroid
LCQ: Leicester Cough Questionnaire
MD: Mean difference
MCID: Minimal clinically important difference
OR: Odds ratios
RCTs: Randomized controlled trials
RR: Risk ratio
SMD: Standardized mean difference
VAS: Visual analogue scale.

## Supplementary Materials

Supplementary Materials Figure S1: risk of bias graph; Figure S2: forest plot of CD4+/CD8+ ratio; Figure S3: forest plot of forced expiratory volume in one second (FEV1); Figure S4: forest plot of effective rate; Figure S5: sensitivity analyses of FEV1 (%) in herbal medicine versus ICS plus bronchodilator; Table S1: GRADE evaluation of herbal medicine versus montelukast; Table S2: GRADE evaluation of herbal medicine versus ICS plus bronchodilators; Table S3: GRADE evaluation of Chinese herbal medicine versus placebo.
