# Supplementary material for: Herbal Medicine for Adult Patients with Cough Variant Asthma: A Systematic Review and Meta-Analysis
Source: Evid Based Complement Alternat Med. 2021 Mar 2;2021:5853137. doi: 10.1155/2021/5853137 (PMC7943289; doi:10.1155/2021/5853137)
Supplement: Supplementary Materials — Figure S1: risk of bias graph; Figure S2: forest plot of CD4+/CD8+ ratio; Figure S3: forest plot of forced expiratory volume in one second (FEV1); Figure S4: forest plot of effective rate; Figure S5: sensitivity analyses of FEV1 (%) in herbal medicine versus ICS plus bronchodilator; Table S1: GRADE evaluation of herbal medicine versus montelukast; Table S2: GRADE evaluation of herbal medicine versus ICS plus bronchodilators; Table S3: GRADE evaluation of Chinese herbal medicine versus placebo. [file 5853137.f1.pdf]

SUPPLEMENTARY INFORMATION

TITLE: Herbal Medicine for Adult Patients with Cough Variant Asthma: A Systematic Review and Meta-analysis

(*List available Supplementary Information items in the following order*)

Figure S1- *Risk of bias graph*

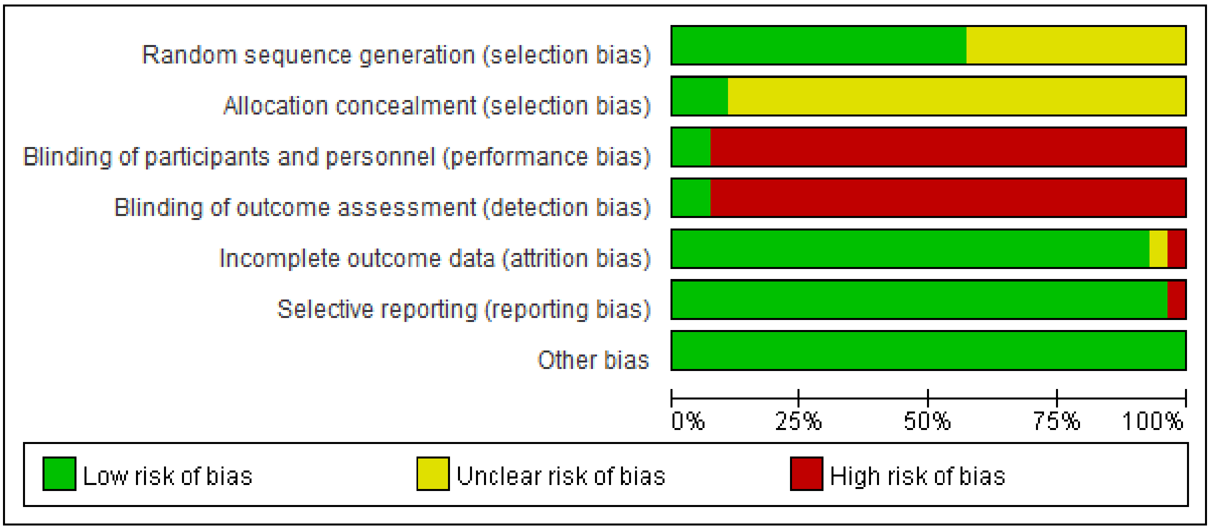

Figure S2- *Forrest plot of CD4+/CD8+ ratio*

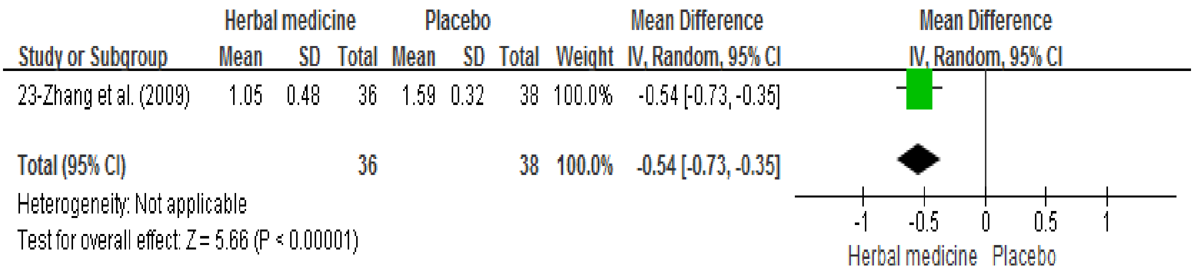

Figure S3- *Forrest plot of Forced Expiratory Volume in One Second (FEV1)*

A Herbal Medicine versus Montelukast

(1) FEV_1 (%)

	Herbal medicine			Montelukast				Mean Difference
Study or Subgroup	Mean	SD	Total	Mean	SD	Total	Weight	IV, Random, 95% CI
27-Zhang et al. (2014)	90.67	4.2	24	87.83	4.6	24	100.0%	2.84 [0.35, 5.33]
**Total (95% CI)**			**24**			**24**	**100.0%**	**2.84 [0.35, 5.33]**

Heterogeneity: Not applicable
Test for overall effect: Z = 2.23 (P = 0.03)

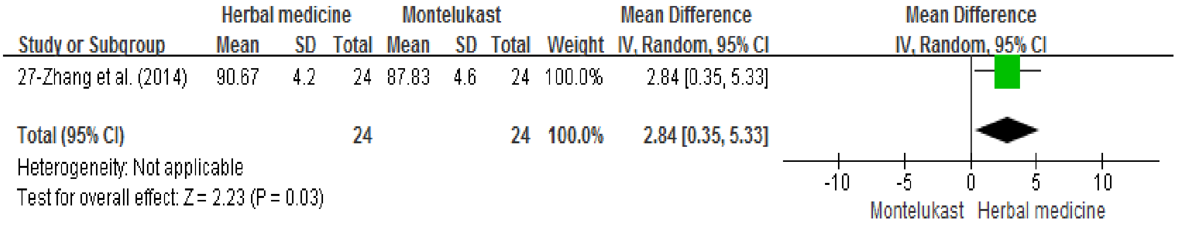

(2) FEV_1 (L)

	Herbal medicine			Montelukast				Mean Difference
Study or Subgroup	Mean	SD	Total	Mean	SD	Total	Weight	IV, Random, 95% CI
31-Kang et al. (2017)	2.98	0.57	50	2.69	0.56	50	100.0%	0.29 [0.07, 0.51]
**Total (95% CI)**			**50**			**50**	**100.0%**	**0.29 [0.07, 0.51]**

Heterogeneity: Not applicable
Test for overall effect: Z = 2.57 (P = 0.01)

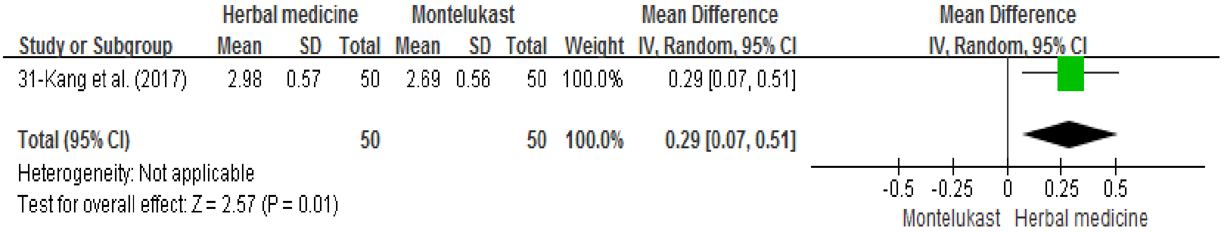

B Herbal Medicine versus ICS plus Bronchodilator

(1) FEV_1 (%)

	Herbal medicine			ICS plus Bronchodilator				Mean Difference
Study or Subgroup	Mean	SD	Total	Mean	SD	Total	Weight	IV, Random, 95% CI
42-Zhao et al. (2015)	89.7	6.9	43	83.6	7.2	40	31.3%	6.10 [3.06, 9.14]
44-Sun et al. (2016)	79.9	4.3	32	76.5	3.3	32	47.8%	3.40 [1.52, 5.28]
46-Lu et al. (2016)	96.55	15.68	40	92.72	12.2	40	11.4%	3.83 [-2.33, 9.99]
49-Lu et al. (2017)	95.16	13.21	30	96.66	13.91	30	9.5%	-1.50 [-8.36, 5.36]
**Total (95% CI)**			**145**			**142**	**100.0%**	**3.83 [1.55, 6.10]**

Heterogeneity: Tau² = 1.90; Chi² = 4.66, df = 3 (P = 0.20); I² = 36%
Test for overall effect: Z = 3.30 (P = 0.0010)

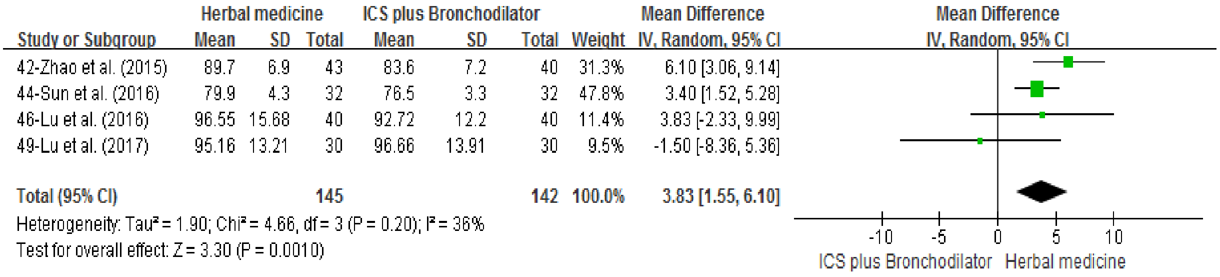

(2) FEV_1 (L)

	Herbal medicine			ICS plus Bronchodilator				Mean Difference
Study or Subgroup	Mean	SD	Total	Mean	SD	Total	Weight	IV, Fixed, 95% CI
40-Xin et al. (2015)	2.52	0.31	60	2.53	0.3	58	100.0%	-0.01 [-0.12, 0.10]
**Total (95% CI)**			**60**			**58**	**100.0%**	**-0.01 [-0.12, 0.10]**

Heterogeneity: Not applicable
Test for overall effect: Z = 0.18 (P = 0.86)

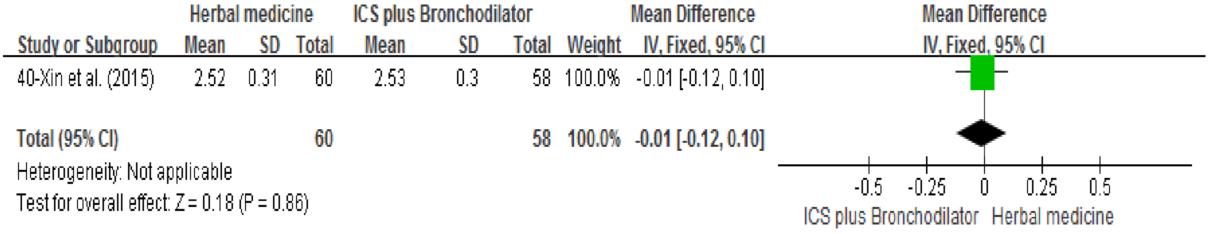

Figure S3 – A and B, Forrest plot of Forced Expiratory Volume in One Second (FEV1).

Figure S4- *Forrest plot of effective rate*

A Herbal Medicine versus Placebo

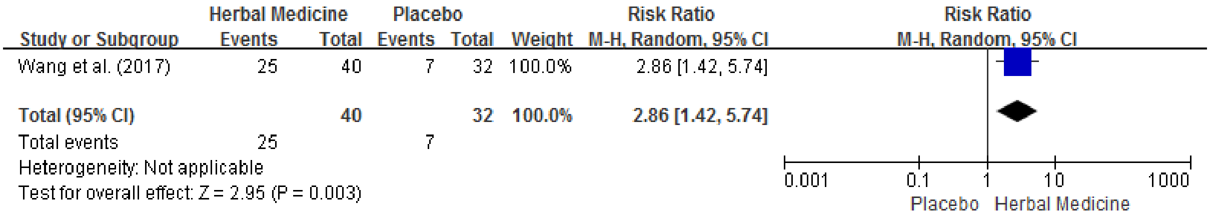

B Herbal Medicine versus Montelukast

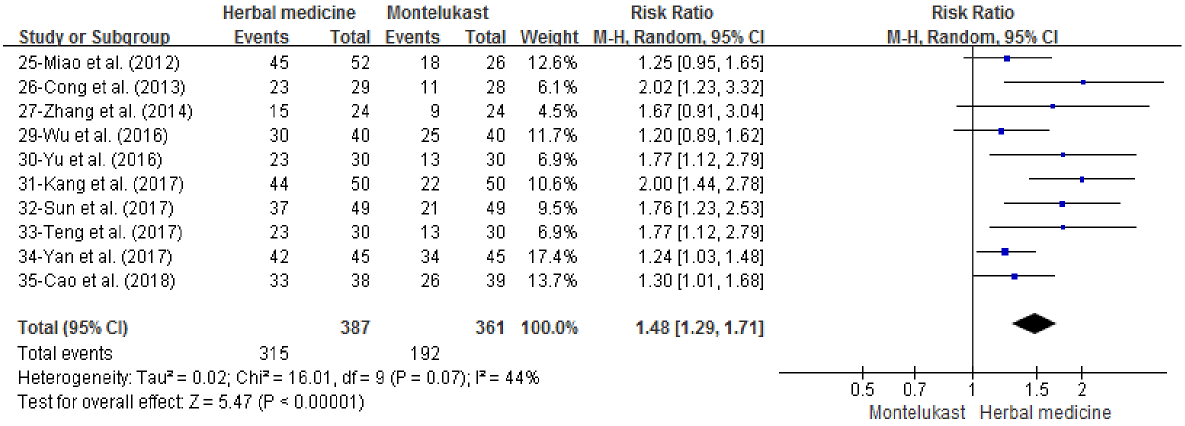

C Herbal Medicine versus ICS plus Bronchodilator

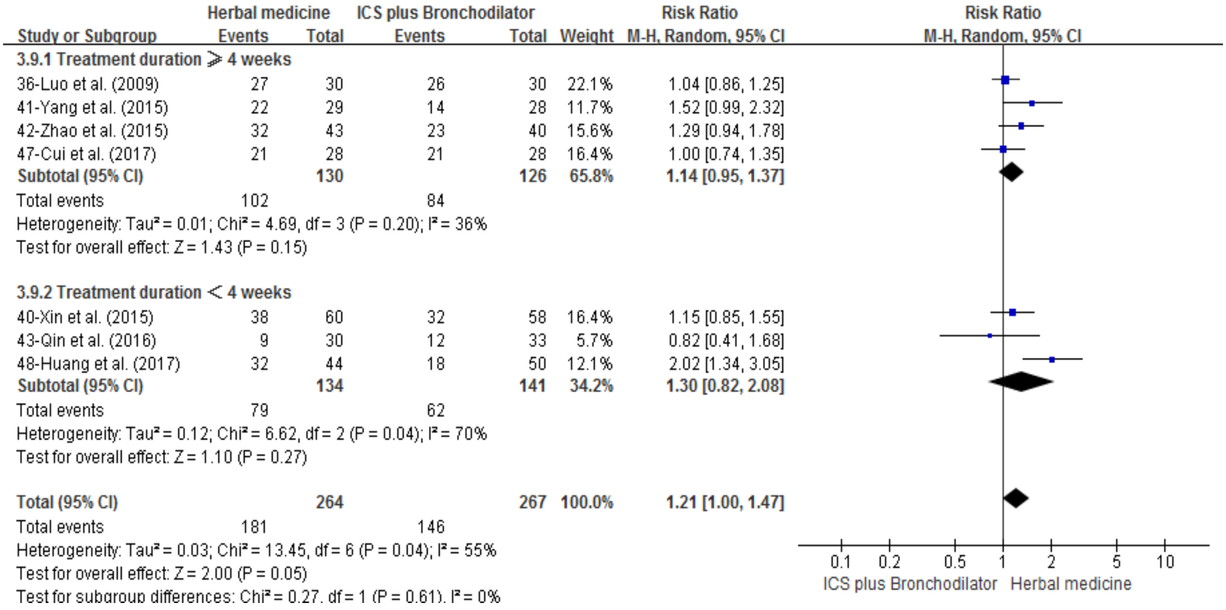

Figure S4 – A, B and C, Forrest plot of effective rate.

Figure S5- *Sensitivity analyses of FEV1 (%) in Herbal Medicine versus ICS plus Bronchodilator*

	Herbal Medicine			ICS plus Bronchodilator				Mean Difference
Study or Subgroup	Mean	SD	Total	Mean	SD	Total	Weight	IV, Random, 95% CI
42-Zhao et al. (2015)	89.7	6.9	43	83.6	7.2	40	48.6%	6.10 [3.06, 9.14]
46-Lu et al. (2016)	96.55	15.68	40	92.72	12.2	40	27.4%	3.83 [-2.33, 9.99]
49-Lu et al. (2017)	95.16	13.21	30	96.66	13.91	30	24.0%	-1.50 [-8.36, 5.36]
**Total (95% CI)**			**113**			**110**	**100.0%**	**3.66 [-0.58, 7.89]**

Heterogeneity: Tau² = 7.19; Chi² = 4.03, df = 2 (P = 0.13); I² = 50%

Test for overall effect: Z = 1.69 (P = 0.09)

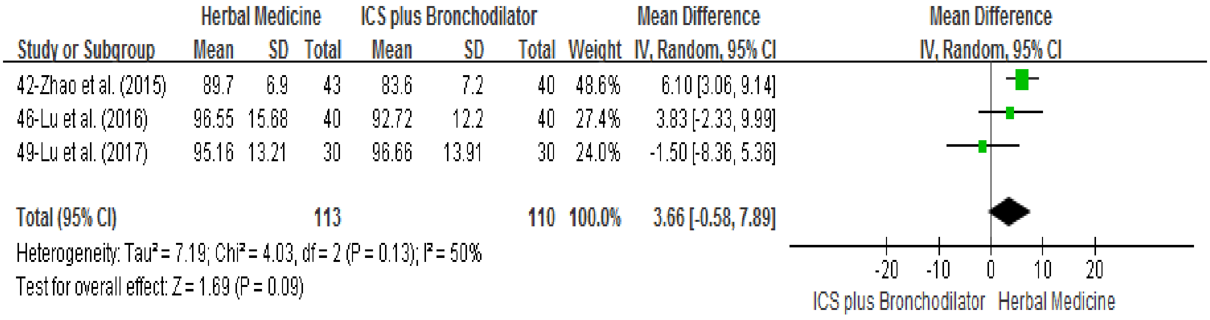

Table S1- *GRADE evaluation of Herbal Medicine vs. Montelukast*

**Outcome**	**Absolute Effect**		**Relative Effect (95% CI)<br>No. of Participants & Studies**	**Certainty of the Evidence (GRADE)**
	With CHM	Without CHM		
LCQ<br>Scale from: 3 to 21[b]<br>Treatment duration:<br>Range 2 to 4 weeks	**15.31**	**13.21**	**MD** 2.38<br>(1.32, 3.44)<br><br>Based on data from 422 patients in 6 studies	⊕⊕○○<br>LOW[1,2]
	Average difference: 2.38 points higher<br>(95% CI: 1.32 higher to 3.44 higher)			
CSS<br>Scale from: 0 to 5[a]<br>Treatment duration:<br>Range 2 to 8 weeks	—		**SMD -0.80**<br>(-1.08, -0.51)<br><br>Based on data from 454 patients in 6 studies	⊕⊕⊕○<br>LOW[1,2]
FEV_1 (L)<br>Treatment duration:<br>Range 4 weeks	**2.98**	**2.69**	**MD 0.29**<br>(0.07 to 0.51)<br><br>Based on data from 100 patients in 1 study	⊕⊕○○<br>LOW [1,3]
	Average difference: 0.29 litres higher<br><br>(95% CI: 0.07 higher to 0.51 higher)			
VAS<br>Scale from: 0 to 10[a]<br>Treatment duration:<br>Range 4 weeks	**2.61**	**3.95**	**MD -1.34**<br>(-1.82, -0.86)<br><br>Based on data from 77 patients in 1 study	⊕⊕○○<br>LOW [1,3]
	Average difference: 1.34 points lower<br><br>(95% CI: 1.82 lower to 0.86 lower)			
Effective rate<br>Treatment duration:<br>Range 2 to 8 weeks	**78**<br>per 100	**50**<br>per 100	**RR 1.48**<br>(1.29 to 1.71)<br><br>Based on data from 748 patients in 10 studies	⊕⊕○○<br>MODERATE [1]
	Difference: 28 more per 100 patients<br><br>(95% CI: 15 to 38 more per 100 patients)			

The risk in the intervention group (and its 95% confidence interval) is based on the assumed risk in the comparison group and the relative effect of the intervention (and its 95% CI). Abbreviations: CHM, Chinese herbal medicine; CI, confidence interval; CSS, Cough symptom score; FEV_1, forced expiratory volume-one second; LCQ, Leicester Cough Questionnaire; MD, mean difference; RR, risk ratio; SMD, standardised mean difference; VAS, Visual Analogue Scale.

a. Higher scores indicate more symptoms; b. Higher scores indicate less symptoms.

1. Unclear sequence generation and allocation concealment. Lack of blinding of participants and personnel;
2. Considerable statistical heterogeneity;
3. Small sample size.

Table S2- *GRADE evaluation of Herbal Medicine vs. ICS plus Bronchodilators*

<table>
<tr><th rowspan="2">Outcome</th><th colspan="2">Absolute Effect</th><th rowspan="2">Relative Effect (95% CI)
No. of Participants & Studies</th><th rowspan="2">Certainty of the Evidence (GRADE)</th></tr>
<tr><td>With CHM</td><td>Without CHM</td></tr>
<tr><td rowspan="2">LCQ
Scale from: 3 to 21[b]
Treatment duration: Range 2 weeks</td><td>15.95</td><td>14.93</td><td rowspan="2">MD 1.02
(0.01, 2.05)

Based on data from 62 patients in 1 study</td><td rowspan="2">⊕⊕○○
LOW[1,3]</td></tr>
<tr><td colspan="2">Average difference: 1.02 points higher

(95% CI: 0.01 high to 2.05 higher)</td></tr>
<tr><td>CSS
Scale from: 0 to 5[a]
Treatment duration: Range 4 to 8 weeks</td><td colspan="2">-</td><td>SMD -0.25 SD
(-0.89, 0.39)

Based on data from 369 patients in 5 studies</td><td>⊕⊕○○
LOW [1,2]</td></tr>
<tr><td rowspan="2">FEV_1 (%)
Treatment duration: Range 2 to 12 weeks</td><td>90.33</td><td>87.37</td><td rowspan="2">MD 3.83
(1.55 to 6.10)

Based on data from 287 patients in 4 studies</td><td rowspan="2">⊕⊕○○
LOW [1,3]</td></tr>
<tr><td colspan="2">Average difference: 3.83% higher

(95% CI: 1.55% higher to 6.10% higher)</td></tr>
<tr><td rowspan="2">VAS
Scale from: 0 to 10[a]
Treatment duration: Range 2 to 4 weeks</td><td>2.78</td><td>3.48</td><td rowspan="2">MD -0.67
(-1.80, 0.46)

Based on data from 281 patients in 4 studies</td><td rowspan="2">⊕○○○
VERY LOW [1,2,3]</td></tr>
<tr><td colspan="2">Average difference: 0.67 points lower

(95% CI: 1.80 lower to 0.46 higher)</td></tr>
<tr><td rowspan="2">Effective rate
Treatment duration: Range 2 to 8 weeks</td><td>69
per 100</td><td>55
per 100</td><td rowspan="2">RR 1.21
(1.00 to 1.47)

Based on data from 531 patients in 7 studies</td><td rowspan="2">⊕⊕○○
LOW [1,2]</td></tr>
<tr><td colspan="2">Difference: 14 more per 100 patients

(95% CI: 0 to 26 more per 100 patients)</td></tr>
</table>

The risk in the intervention group (and its 95% confidence interval) is based on the assumed risk in the comparison group and the relative effect of the intervention (and its 95% CI). Abbreviations: CHM, Chinese herbal medicine; CI, confidence interval; CSS, Cough symptom score; FEV_1, forced expiratory volume-one second; LCQ, Leicester Cough Questionnaire; MD, mean difference; RR, risk ratio; SMD, standardised mean difference; VAS, Visual Analogue Scale.

a. Higher scores indicate more symptoms; b. Higher scores indicate less symptoms.

1. Unclear sequence generation and allocation concealment. Lack of blinding of participants and personnel;
2. Considerable statistical heterogeneity;
3. Small sample size.

Table S3- *GRADE evaluation of Herbal Medicine vs. Placebo*

Outcome	Absolute Effect		Relative Effect (95% CI) No. of Participants & Studies	Certainty of the Evidence (GRADE)
	With CHM	Without CHM		
CSS<br>Scale from: 0 to 5[a]<br>Treatment duration: Range 2 weeks	**2.35**	**3.5**	**MD -1.15**<br>(-1.67, -0.63)<br><br>Based on data from 72 patients in 1 study	⊕⊕⊕○<br>MODERATE [1]
	Average difference: 1.15 points lower<br><br>(95% CI: 1.67 lower to 0.63 lower)			
VAS<br>Scale from: 0 to 10[a]<br>Treatment duration: Range 2 weeks	**3.08**	**4.84**	**MD -1.76**<br>(-2.66, -0.86)<br><br>Based on data from 72 patients in 1 study	⊕⊕⊕○<br>MODERATE [1]
	Average difference: 1.76 points lower<br><br>(95% CI: 2.66 lower to 0.86 lower)			
CD4+/CD8+ ratio<br>Treatment duration: Range 8 weeks	**1.05**	**1.59**	**MD -0.54**<br>(-0.73, -0.35)<br><br>Based on data from 74 patients in 1 study	⊕⊕⊕○<br>MODERATE [1]
	Average difference: 0.54 lower<br><br>(95% CI: 0.73 lower to 0.35 lower)			
Effective rate<br>Treatment duration: Range 2 weeks	**63** per 100	**22** per 100	**RR 2.86**<br>(1.42 to 5.74)<br><br>Based on data from 72 patients in 1 study	⊕⊕⊕○<br>MODERATE [1]
	Difference: 41 more per 100 patients<br><br>(95% CI: 9 to 100 more per 100 patients)			

The risk in the intervention group (and its 95% confidence interval) is based on the assumed risk in the comparison group and the relative effect of the intervention (and its 95% CI). Abbreviations: CHM, Chinese herbal medicine; CI, confidence interval; CSS, Cough symptom score; MD, mean difference; RR, risk ratio; VAS, Visual Analogue Scale.

a. Higher scores indicate more symptoms.

1. Small sample size.
